# Serovar-level identification of bacterial foodborne pathogens from full-length 16S rRNA gene sequencing

**DOI:** 10.1128/msystems.00757-23

**Published:** 2024-02-06

**Authors:** Dmitry Grinevich, Lyndy Harden, Siddhartha Thakur, Benjamin Callahan

**Affiliations:** 1 Department of Population Health and Pathobiology, College of Veterinary Medicine, North Carolina State University, Raleigh, North Carolina, USA; 2 Bioinformatics Research Center, North Carolina State University, Raleigh, North Carolina, USA; Dalhousie University, Halifax, Nova Scotia, Canada

**Keywords:** serotyping, food-borne pathogens, *Salmonella*, *Escherichia coli*, 16S rRNA, long-read sequencing

## Abstract

The resolution of variation within species is critical for interpreting and acting on many microbial measurements. In the key foodborne pathogens *Salmonella* and *Escherichia coli*, the primary subspecies classification scheme used is serotyping: differentiating variants within these species by surface antigen profiles. Serotype prediction from whole-genome sequencing (WGS) of isolates is now seen as comparable or preferable to traditional laboratory methods where WGS is available. However, laboratory and WGS methods depend on an isolation step that is time-consuming and incompletely represents the sample when multiple strains are present. Community sequencing approaches that skip the isolation step are, therefore, of interest for pathogen surveillance. Here, we evaluated the viability of amplicon sequencing of the full-length 16S rRNA gene for serotyping *Salmonella enterica* and *E. coli*. We developed a novel algorithm for serotype prediction, implemented as an R package (Seroplacer), which takes as input full-length 16S rRNA gene sequences and outputs serovar predictions after phylogenetic placement into a reference phylogeny. We achieved over 89% accuracy in predicting *Salmonella* serotypes on *in silico* test data and identified key pathogenic serovars of *Salmonella* and *E. coli* in isolate and environmental test samples. Although serotype prediction from 16S rRNA gene sequences is not as accurate as serotype prediction from WGS of isolates, the potential to identify dangerous serovars directly from amplicon sequencing of environmental samples is intriguing for pathogen surveillance. The capabilities developed here are also broadly relevant to other applications where intraspecies variation and direct sequencing from environmental samples could be valuable.

**IMPORTANCE:**

In order to prevent and stop outbreaks of foodborne pathogens, it is important that we can detect when pathogenic bacteria are present in a food or food-associated site and identify connections between specific pathogenic bacteria present in different samples. In this work, we develop a new computational technology that allows the important foodborne pathogens *Escherichia coli* and *Salmonella enterica* to be serotyped (a subspecies level classification) from sequencing of a single-marker gene, and the 16S rRNA gene often used to surveil bacterial communities. Our results suggest current limitations to serotyping from 16S rRNA gene sequencing alone but set the stage for further progress that we consider likely given the rapid advance in the long-read sequencing technologies and genomic databases our work leverages. If this research direction succeeds, it could enable better detection of foodborne pathogens before they reach the public and speed the resolution of foodborne pathogen outbreaks.

## INTRODUCTION

Foodborne illnesses caused by bacterial pathogens are a major cause of hospitalizations and deaths throughout the United States, and the subspecies classification of different serovars within important enteric pathogens is important for surveillance and intervention. *Salmonella* species are the leading cause of hospitalizations according to Centers for Disease Control and Prevention (CDC) data, accounting for over 23,000 annual hospitalizations and over 450 annual deaths (1). Treatment of salmonellosis has been estimated to cost over 2.5 billion dollars annually (2). The “big six” *Escherichia coli* serotypes, the primary pathogenic *E. coli* causing foodborne illness, are responsible for nearly 170,000 cases of foodborne illnesses in the United States annually (3). The burden caused by foodborne pathogens calls for modern surveillance tools that can rapidly identify these pathogens, prevent their introduction into the food system and people’s bodies, and rapidly trace outbreaks back to their source. However, many pathogen species can also be commensal organisms and occupy a normal environmental niche in the mammalian gut (4, 5). Upward of 90% of *E. coli* strains are predicted to be commensal; we need ways to distinguish which strains are dangerous for human health and which are not (4). The most common subspecies classification of both *Salmonella* and *E. coli* is the division into serovars based on their antigenic properties. The vast majority of illness caused by *Salmonella* derives from a small number of serovars including Enteritidis, Newport, Typhimurium, Monophasic Typhimurium, and Javiana. Similar patterns hold in pathogenic *E. coli*, with, for example, serovar O157:H7 being responsible for a large fraction of serious disease cases.

In clinical and applied microbiology, species-level identification has become standard, but increasingly, we recognize the importance of subspecies classifications in order to effectively act on microbiological measurements. A wide variety of subspecies typing methods are employed across different contexts, including methods such as biological testing for antigenic gene features, molecular typing methods including PCR and microarray marker gene analyses, and computational typing approaches using whole-genome sequencing (WGS) data (6–10). Serotyping through traditional microbiology methods is the most widely used method for subspecies classification of enteric pathogens but is labor-intensive and time-consuming. Traditional serotyping requires lab access to many antisera which are necessary for testing for a variety of possible surface antigens. In the past decade, sequencing has played an increasingly important role in subspecies identification, and it is now common to perform computational serotyping using WGS data from isolates of enteric pathogens. Popular tools for computational serotyping include the *Salmonella in Silico* Serotyping Resource (SISTR) and SeqSero2 (8, 9). In *E. coli*, tools such as SerotypeFinder and ECTyper have been developed for computational O and H antigen prediction (11, 12). Computational serotyping from WGS data achieves high accuracy, but important limitations remain including the cost of WGS and the need to isolate and culture individual microbes from potentially complex samples to obtain the necessary genetic material.

Here, we develop an approach using sequences of the full-length 16S rRNA gene sequences to classify *Salmonella* and *E. coli* to the serovar level. If serovar-level resolution could be obtained from 16S rRNA gene sequences alone, then it may be possible to improve pathogen surveillance by applying 16S rRNA gene amplicon sequencing directly to complex food-associated samples without requiring an isolation and culture step. Furthermore, the recent progress in long-read sequencing platforms has made amplicon sequencing of the full-length 16S rRNA gene increasingly affordable, accessible, and accurate. Serovar prediction from a marker gene is fundamentally different from prediction from WGS data, as the 16S rRNA gene can only provide phylogenetic information, not direct information on the O and H antigen gene sequences. Thus, we leverage a reference database of *Salmonella* and *E. coli* strains with known serotypes, fully enumerated complements of their multicopy 16S rRNA genes, and an associated high-quality phylogeny linking the reference strains. We evaluated the viability of serotyping enteric bacteria using full-length 16S rRNA gene sequencing data and developed user-friendly software for serovar prediction from such data in *Salmonella* and *E. coli*. Although our focus here is on *Salmonella* and *E. coli*, the methods we develop are generalizable to many other bacterial taxa for which sufficient numbers of sequenced genomes and appropriate subspecies classification schema are available.

## MATERIALS AND METHODS

### Curation of reference database

Complete tables of all available reference assemblies were downloaded from the GenomeTrakr database (https://www.ncbi.nlm.nih.gov/pathogens/) for both *Salmonella enterica* (10 July 2022) and *E. coli* (5 September 2022). Initial filtering was done by choosing all assemblies with assembly completion thresholds of “chromosome” or “complete genome,” as determined by National Center for Biotechnology Information (NCBI). We also required that all reference assemblies contained exactly seven full-length 16S rRNA gene sequences, and whole-genome computational serovar prediction yielded a complete and unambiguous serovar. For *Salmonella* entries, we removed reference assemblies that failed to receive an O-antigen assignment, identified as O-antigen assignments marked “-” in the output of SISTR. For *E. coli* entries, we removed reference assemblies that were missing either an O- or H-antigen assignment, identified as assignments marked as “-” in the output of ECTyper. The final reference database(s) was stored as a FASTA file, with 7 16S rRNA gene sequences for each reference genome labeled in the FASTA file by the original assembly, 16S rRNA allele number, and computational serovar assignment.

### Serovar assignment to reference genomes

*In silico* serovar prediction on *Salmonella* assemblies was done using the SISTR ver. 1.1.1 (available at https://github.com/phac-nml/sistr_cmd). SISTR was run using the sistr_cmd command following recommended standard parameters (sistr_cmd -o json -qc). Serovar prediction on *E. coli* was performed using the ECTyper tool (available at https://github.com/phac-nml/ecoli_serotyping). Analysis was run using the ectyper command with default options, and a presketched mash archive was downloaded on 19 August 2021 from https://gembox.cbcb.umd.edu/mash/refseq.genomes.k21s1000.msh. The Typhimurium and Monophasic Typhimurium serovars in *Salmonella* form a single-distinct clade that cannot be distinguished using core genes or 16S rRNA gene sequences. Thus, we chose to merge them in our reporting and classify them as a single group labeled “Typhimurium/Monophasic Typhimurium.”

### Construction of reference phylogenetic trees

Core genes for each species were defined as those genes present in every reference genome. We identified a pool of 100 core genes for *Salmonella* and 101 core genes for *E. coli* species. MAFFT (ver. 7.490, available at https://mafft.cbrc.jp/alignment/software/) was used to generate multiple sequence alignments for each core gene (parameters: --retree 2—inputorder). The concatenated alignments of all core genes were then used to infer the phylogeny linking the reference strains using the RAxML-ng tool (ver. 1.1.0, available at https://github.com/amkozlov/raxml-ng) with the raxml-ng command (parameters: –model GTR + G) (13, 14). The final tree was stored in newick format for input into downstream phylogenetic placement analysis.

### Serovar assignment

Our serovar assignment algorithm takes as input a complete set of the seven 16S rRNA gene sequences from an isolate of *Salmonella* or *E. coli* given, corresponding to the seven copies of the 16S rRNA gene present in both species’ genomes. Query sequences are first aligned to a prealigned 16S allele reference containing all of our reference 16S rRNA gene sequences using the mafft command from MAFFT (parameters: --add --keeplength). The correct relative ordering of the query 16S rRNA sequences and the 16S rRNA gene sequences retrieved from the reference assembly is unknown *a priori*. So, at this stage, the set of reference 16S alleles is rearranged to optimally match the query. More precisely, for each reference assembly in the database, its set of seven 16S alleles is compared against the seven query sequences, and the reference alleles are rearranged into the order that minimizes total nucleotide mismatches to the query. Query sequences and rearranged reference 16S rRNA gene sequences are concatenated and stored as FASTA input files for phylogenetic placement through an evolutionary placement algorithm (EPA-ng; ver. 0.3.8, available at https://github.com/Pbdas/epa-ng) (15, 16). The query is phylogenetically placed into the reference phylogeny using epa-ng command (parameters: –filter-max 100) and a model parameter specification evaluated from RAXML-ng using the evaluate command (raxml-ng --evaluate --msa $REF_MSA --tree $TREE --prefix info --model GTR + G + F). After query sequence placement, all placements reported by EPA-NG within the 99.9% likelihood weight ratio range are considered candidate placements.

**Fig 1:**
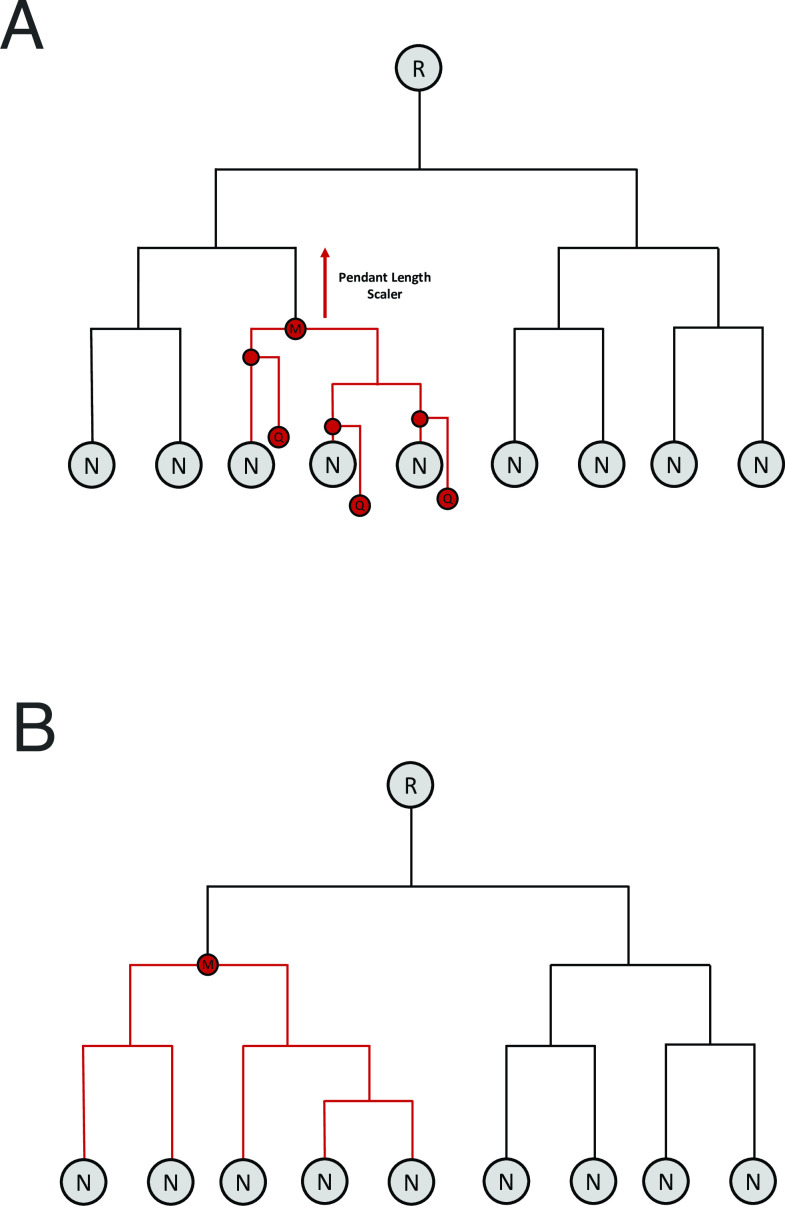
Proposed serovar assignment algorithm. (**A**) Queries (Q) consisting of the full complement of seven 16S rRNA gene sequences are phylogenetically placed into a reference phylogenetic tree consisting of terminal nodes (N) and root (R). Typically, multiple potential placements are predicted, from which an initial MRCA (M) is constructed. The maximum pendant length (i.e., the length of the new branch inserted into the tree to place the query) is recorded. The MRCA is then adjusted upward toward the root of the tree by the maximum pendant length times a predetermined scalar value of order 1, yielding the final MRCA shown in panel **B**. A serovar assignment is made if more than a threshold fraction (by default 0.5) of the reference entries contained in the phylogenetic clade defined by the final MRCA are of the same serovar.

Candidate placements of the query into the reference phylogeny are assigned to either the immediately distal or immediately proximal node to the insertion site of the pendant branch, whichever was closer to the insertion point. The initial most recent common ancestor (MRCA) is defined as the MRCA of the nodes assigned to each candidate placement. In order to account for uncertainty in the phylogenetic placements, the initial MRCA is then moved higher in the tree (Fig. 1 ) in a step we call “pendant length adjustment.” A branch length K is defined as the maximum pendant length from the candidate placements multiplied by an arbitrary scalar value. The phylogenetic tree is traversed from the initial MRCA toward the root by branch length *K*, and the closest node (distal or proximal) to this position is defined as the pendant-adjusted MRCA. If all candidate placements have short pendant lengths (suggestive of high confidence in the insertion site), it is possible that the MRCA will not change during pendant length adjustment. Here, we used a pendant length multiplier value of 1.5 based on a data-driven evaluation in *Salmonella* and *E. coli*.

The set of reference assemblies contained in the clade defined by the pendant-adjusted MRCA is considered the set of “hits” of our algorithm. A specific serovar assignment is made if greater than some threshold fraction *T* of the hits are of the same serovar. By default, our computational tool sets *T* = 50% and reports serovar as indeterminate if no serovar exceeds 50% of the hits. However, users can also obtain a complete report of all serovars included in the final hits and their associated percentages.

### The Seroplacer R package

The serovar prediction algorithm described above is available as an R package available from Github (https://github.com/Dogrinev/Seroplacer). The required input for classification by Seroplacer is a FASTA file containing the seven 16S rRNA gene sequences from a single-bacterial isolate, matching the total copy number found in *Salmonella and E. coli*. The current implementation of the Seroplacer package requires that all seven copies of the 16S rRNA genes are present for downstream analysis. Reference data files for *Salmonella* and *E. coli*, including the phylogenetic tree and 16S rRNA gene sequence files, are included with the package. The target species for classification is then simply provided as a string to the relevant package functions. The Data_Preparation function reformats the sequence data from the user input FASTA file for use in the placement algorithm. The serovar prediction method employed by the algorithm depends on two external command line tools, EPA-NG and MAFFT (15–17), which are executable through provided wrapper functions in the package, mafft_wrap and epa_ng_wrap. The 16S rRNA gene reference sequence data used for serovar classification are stored in non- and prealigned FASTA files. The reference phylogenetic trees used by the package are stored in newick tree format and included with the package during download. After phylogenetic placement through the core function Clade_Hit_Finder_Pendant, the software outputs a table containing all serovars identified in the final phylogenetic clade predicted, the associated percentages of each serovar, and a serovar prediction. A vignette included in the package provides an example classification using test data.

### Environmental and sample isolates

We obtained 10 *S. enterica* isolates from previously collected stocks obtained during pathogen surveillance by the Thakur Molecular Epidemiology Laboratory at North Carolina State University. These isolates covered a variety of isolation sources including human feces, vegetative buffer, sheep feces, lairage swabs, caprine feces, and cecal content. We obtained 27 animal samples from routine retail meat surveillance testing that contained a variety of microbes with a fraction of samples containing *E. coli*. Meat samples were enriched in buffered peptone water and stored for sequencing analysis.

### Environmental isolate sequencing

Genomic DNA for the bacterial isolates was extracted using the DNeasy PowerLyser Microbial Kit following manufacturer protocols (Qiagen). DNA concentrations were measured using NanoDrop Spectrophotometry (Thermo Fisher Scientific) and normalized to contain approximately 100 ng/µL and 30 µL of bacterial DNA. 16S rRNA gene sequencing libraries for 10 *Salmonella* isolates and 27 animal-sourced isolates were prepared using the LoopSeq pipeline from Loop Genomics following manufacturer protocols (loopgenomics.com). Loop Genomics 16S rRNA gene primers were used to amplify the near full-length gene (Forward: AGAGTTTGATCMTGGC and Reverse: TACCTTGTTACGACTT). Isolate samples were prepared to measure 1,000–1.5 kb molecules per sample, and environmental samples were prepared to measure 50,000–1.5 kb molecules per sample. Amplicon sequencing data were quality filtered, denoised, and analyzed using primarily the standard dada2 workflow (available at https://github.com/benjjneb/dada2) with modified parameters designed to improve sensitivity when analyzing LoopSeq data (18). Adjustments included changes to the filterAndTrim function (maxEE = 0.5) and the dada function (OMEGA_A = 1e-10 and DETECT_SINGLETONS = TRUE). Denoised 16S rRNA gene sequences were assembled into sets of seven 16S rRNA gene alleles and converted to FASTA file format for serovar analysis. 16S rRNA gene query sequences were inputted into the serovar placement algorithm described previously, and final serovar predictions were recorded.

### Computational analyses and statistics

Statistical analyses and visualizations were performed using R statistical computing software (ver. 4.2.1) and RStudio (ver. 2022.07.1+554). Phylogenetic distance calculations throughout this work are all performed using the “ape” package (ver. 5.6-2) in R.

## RESULTS

### Curation of reference sequence data and phylogenetic trees

We downloaded a list of 1,787 *Salmonella* assemblies with “chromosome” or “complete genome” quality from the GenomeTrakr database (https://www.ncbi.nlm.nih.gov/pathogens/) on 10 July 2022. Of these, 1,700 reference assemblies contained exactly seven full-length 16S rRNA gene sequences and a complete set of *Salmonella* core genes. We used the SISTR serotyping method to assign serovars to each reference assembly (9). Eighty-two entries were not assigned an O-antigen label and were removed, leaving 1,618 assemblies in our final *Salmonella* reference database representing 155 unique serotypes. A phylogenetic tree was inferred from a concatenated alignment of the *Salmonella* core genes using RaXML and 20 bootstrapped replicates (Materials and Methods). Our phylogeny recapitulated known major features of the *Salmonella* phylogeny, including reconstructing known clade groups (I–V). All seven full-length 16S rRNA gene sequences were extracted from each reference assembly and organized into two reference FASTA files, one in which the sequences are unaligned and another in which they are aligned using MAFFT (Materials and Methods). The ID line of the reference fasta files was formatted with the assembly identifier, the 16S rRNA gene copy number (1–7), and the assigned serotype. We followed the same process to develop a reference database for *E. coli*, yielding a total of 2,791 assemblies representing 692 unique serotypes in our *E. coli* reference database.

### 16S rRNA gene profiles are on average more similar within serovars

**Fig 2:**
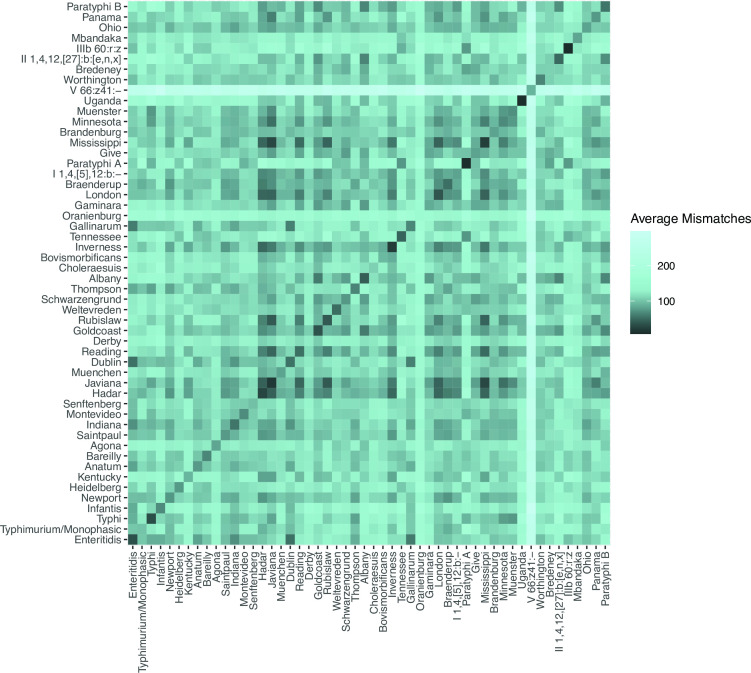
Average dissimilarity between the 16S rRNA gene profiles of *Salmonella* serovars. Pairwise dissimilarities were defined as the number of nucleotide mismatches between alignments of the full set of 16S rRNA genes (the 16S rRNA gene profile) of *Salmonella* assemblies, after optimally rearranging the profiles relative to one another (Materials and Methods). The dissimilarity between assemblies of the same serovar was lower on average than between different serovars (darker colors on the diagonal). However, some pairs of serovars had 16S rRNA gene profiles that were as or more similar than the profiles within those serovars (dark cells off the diagonal).

We performed an initial evaluation of whether 16S rRNA gene sequences carry sufficient signal to differentiate between serovars by comparing the average dissimilarity in 16S rRNA gene profiles between and within *Salmonella* serovars. For clarity, the “16S rRNA gene profile” refers to the full complement of 16S rRNA gene alleles from a given genome, in arbitrary order. Here, it means that 16S rRNA gene profiles consist of seven sequences, as both *Salmonella* and *E. coli* have seven copies of the 16S rRNA gene in their genomes. We calculated the number of differing nucleotide sites between alignments of the 16S rRNA gene allele profile present in each *Salmonella* genome (Materials and Methods) and then averaged these pairwise nucleotide dissimilarities between and within each serovar with at least four entries in our reference database (Fig. 2).

*Salmonella* strains of the same serovar have, on average, lower dissimilarity across their 16S rRNA gene profile than do *Salmonella* strains of different serovars (darker colors along diagonal, Fig. 2), but not always. The average number of nucleotide mismatches is typically greater than 100 (>0.1% dissimilarity) when comparing the 16S rRNA gene profiles from different *Salmonella* serovars. Profiles from same-serovar strains show lower numbers of nucleotide mismatches, with typical values in the 30–50 range (<0.05% dissimilarity). The significantly higher similarity of 16S rRNA gene profiles from same-serovar strains (*P* = 2.2e−16, Wilcoxon rank sum test) suggests that 16S rRNA gene sequences may carry enough signal to make serovar assignments in some cases. However, we also observed low values (<0.05% dissimilarity) between some different pairs of serovars (dark colors off the diagonal, Fig. 2). Thus, 16S rRNA gene profile similarity alone may not always provide sufficient resolution to discriminate serovars. *E. coli* had similar overall patterns. Same-serovar *E. coli* strains showed average nucleotide mismatch values in the 40–70 range, with higher dissimilarity usually, but not always when comparing across serovars (Fig. S1).

### *Salmonella* serovars are phylogenetically coherent but sometimes polyphyletic

**Fig 3:**
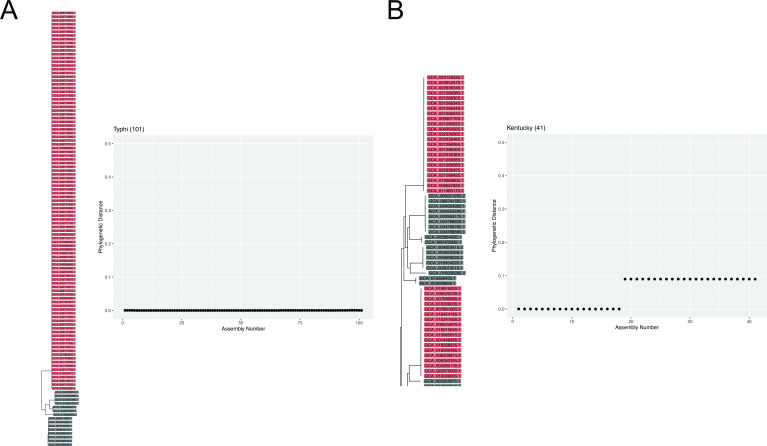
Monophyletic and polyphyletic *Salmonella* serovars. (**A**) Phylogenetic distances between an example Typhi assembly and all other Typhi assemblies in our *Salmonella* reference database. The portion of the phylogenetic tree containing all Typhi assemblies shows they are closely related and form a coherent clade. (**B**) Phylogenetic distances between an example Kentucky assembly and all other Kentucky assemblies in our *Salmonella* reference database. Kentucky strains form two distinct clades within the phylogenetic tree. Some assemblies in the reference phylogeny were removed in panel B for visual clarity.

All large *Salmonella* serovars (20+ assemblies in our reference database) were coherently phylogenetically organized but sometimes into more than one clade. The three largest serovars in our phylogenetic tree, including Enteritidis, Typhi, and Typhimurium, clustered into single clades in which the vast majority of assemblies were assigned to the same serovar (Fig. 3A, Typhi). However, other serovars clustered into two or even three distinct clades, as can be observed in *Salmonella* serovar Kentucky (Fig. 3B). The polyphyletic structure of some serovars complicates the use of simple similarity-based methods for serovar identification but is not in principle problematic for methods based on phylogenetic placement. Out of 21 *E. coli* serovars with the largest representation in our reference database, 14 clustered into single coherent clades, including the three largest serovars (O157:H7, O16:H48, and O25:H4). Polyphyletic organization into two distinct clades was observed in six large serovars, while one large serovar (O11:H25) only partially clustered with a fraction of its assemblies spread throughout the phylogenetic tree.

### Method and software for serovar assignment from 16S rRNA gene profiles

We developed a new algorithm for predicting serovar from 16S rRNA gene profiles based on phylogenetic placement into a reference phylogeny of serovar-labeled reference assemblies (see Materials and Methods for details). In short, we first curated reference databases of high-quality complete genomes with unambiguous serovar assignments for both *Salmonella* and *E. coli*. A core genome phylogeny for each reference database was constructed, and a reference fasta file with entries for each 16S rRNA gene allele from each reference assembly a created. Queries, in the form of complete 16S rRNA gene profiles, were optimally reordered and aligned to the reference 16S rRNA gene profiles. Concatenated alignments were used as input to the maximum-likelihood EPA-NG to place query profiles into the reference phylogenetic tree. Phylogenetic placements and their associated uncertainty were used to define a set of “hits” in the reference database and to make a serovar prediction. The full pipeline is organized as an R software package available at https://github.com/Dogrinev/Seroplacer.

### *In silico* evaluation of serovar assignment from full-length 16s rRNA gene sequencing

We performed an *in silico* evaluation of serovar assignment accuracy using a “leave-one-out” strategy, in which each entry in the reference database was first removed from the database and then used as a query to our algorithm. That is, for each reference assembly, we deleted it from our reference database and then used our method to classify the 16S rRNA gene profile of the deleted reference. We categorized our serovar prediction results into three groups: correct, incorrect, and indeterminate. For correct, a definite serovar assignment was made by our method that matched the ground truth serovar derived from WGS. For incorrect, a definite serovar assignment was made by our method that was different than the WGS serovar prediction. For indeterminate, no serovar assignment was made by our method because no single serovar represented a consensus (i.e., 50% or more) of all leaves within the final clade. We developed two additional evaluation metrics to quantify the performance of our method: serovar accuracy (SA) and query recovery (QR). SA is the fraction of hits under the final clade that correctly matched the serovar of the query. QR is a binary value that is defined as TRUE when the final clade contains the attachment point of the query assembles phylogenetic branch (i.e., would contain the query if the query had not been removed from the reference) and FALSE otherwise.

Our algorithm incorporates uncertainty in phylogenetic placement to broaden the set of references considered as potential “hits” and thus as contributors to the prediction of serovar. In a phylogenetic placement algorithm like EPA-NG, the pendant length represents the length of the newly inserted branch during phylogenetic placement into the existing tree. We considered this as a proxy for the uncertainty in the phylogenetic placement. More precisely, we adjusted the final prediction clade by traversing the reference tree upward by a multiple of the maximum pendant length of qualified phylogenetic placements of the query (Materials and Methods). We tested a range of multipliers for the pendant length adjustment to identify the best value for this algorithm parameter. A larger pendant length adjustment makes the prediction clade more inclusive by shifting the MRCA upward toward the root. An ideal pendant length multiplier value will produce prediction clades that contain the query strain (high QR), while not sacrificing too much specificity in the final result (high SA).

### Algorithm performance in *Salmonella*

**Fig 4:**
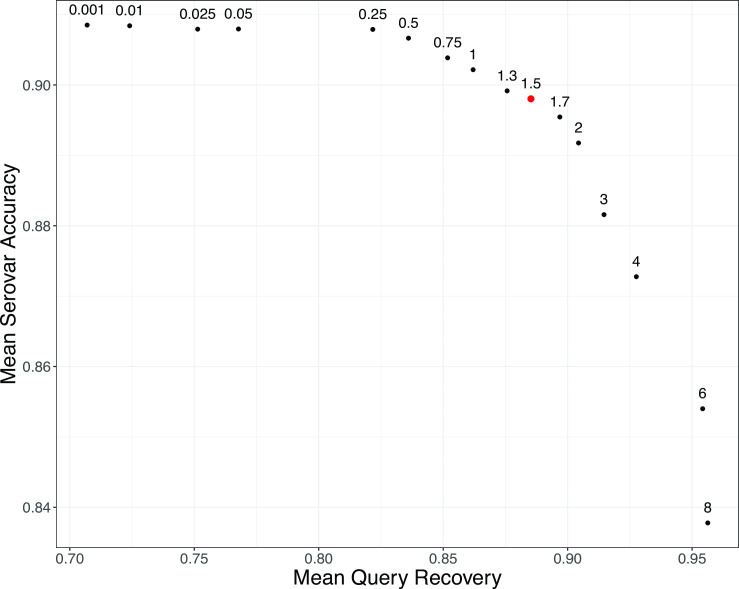
Serovar placement algorithm performance on *Salmonella* test data. For each test query, a final clade of hits was determined by identifying the MRCA of all placements. The summary statistics plotted here are averages across all test queries for each given pendant length multiplier. Mean SA represents the fraction of hits that match the original query serovar averaged across all test queries. Mean QR is the fraction of original test queries that were located within the final calculated clade of hits. Pendant length multiplier values are labeled above the respective data point.

In *Salmonella*, our method had mean SA between 83% and 91% across the range of pendant-length multiplier parameters considered (0.001×–8×) on our test data set consisting of 52 Salmonella serovars with at least four entries in our reference database (Fig. 4). As expected, positive predictive accuracy decreased as the pendant length multiplier was raised because, as the final clade grows in size, an increased number of queries returned indeterminate serovar assignments. However, this decrease in accuracy was minor for multipliers up to ~4×. The fraction of queries recovered increased as the pendant length multiplier was raised since larger final clades were more likely to contain the phylogenetic attachment point of the removed query. From these results, we selected a pendant length multiplier of 1.5 as providing a good balance of mean SA (>89%) and mean QR (>88%, Fig. 4).

**Fig 5:**
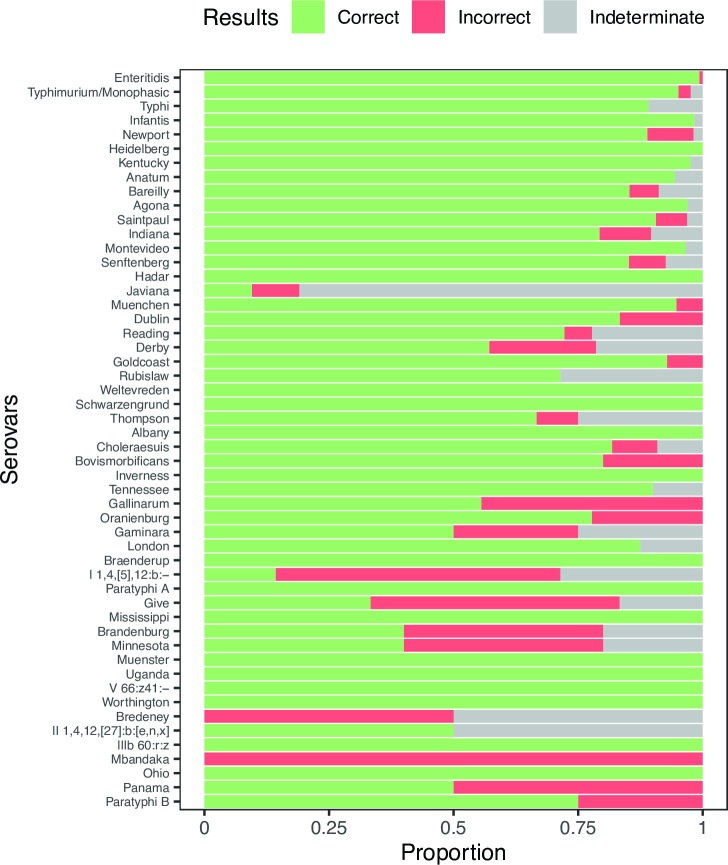
. Serovar prediction accuracy in *Salmonella* varies across serovars. The proportion of query sequences that resulted in correct, incorrect, or indeterminate (Materials and Methods) serovar assignments from the 52 serovars with at least four entries in our *Salmonella* reference database. Serovars are ordered from most representation (Enteritidis, 300 assemblies) to least representation (Paratyphi B; four assemblies).

The accuracy of our serovar assignment method varied among *Salmonella* serovars. The fraction of different serovar assignment outcomes across the 52 serovars present in our test data set was 90.1% correct, 4.5% incorrect, and 5.4% indeterminate, but clear differences among specific serovars were apparent (Fig. 5). Some variation in serovar prediction accuracy is explained by the clear trend of decreased classification accuracy as the number of reference assemblies for a serovar decreased (Fig. 5; Table 1). The largest serovars (Enteritidis: *n* = 300, Typhimurium/Monophasic Typhimurium: *n* = 288, and Typhi: *n* = 101) were all highly predictable (>90% accuracy). The proportion of correct assignments only began to drop below 75% in serovars containing fewer than 10 entries in the reference database. We calculated per-serovar *in silico* assignment outcomes for all serovars with at least four entries in our reference database (Table S1).

**TABLE 1:** Serovar assignment outcomes in *Salmonella* serovars depend on their level of representation in our reference database^*a*^

	Correct	Incorrect	Indeterminate	Number in group
100–300 assemblies	96.1%	2.6%	1.3%	3
20–99 assemblies	89.4%	7.1%	3.5%	13
10–19 assemblies	84.5%	8.6%	6.9%	14
4–9 assemblies	68.0%	9.6%	22.4%	22

^*a*^ Percentage correct, incorrect, and indeterminate rates are averages across serovars stratified into groups by the number of genomes a serovar has in the reference database.

Our method’s performance did not meaningfully degrade when identifying polyphyletic serovars. Some serovars such as Kentucky form two or more distinct clades within the *Salmonella* phylogeny (Fig. 3). In the 20 *Salmonella* serovars with the largest representation in our reference database, we determined that nine did not cluster into one clade. For six of these nine large polyphyletic serovars (Newport, Kentucky, Bareilly, Saintpaul, Montevideo, and Muenchen), we still achieved over an 80% correct prediction rate, suggesting that it is not necessary for each reference to cluster perfectly into one clade in order to make good predictions. For the remaining three large polyphyletic serovars, we observed lower correct prediction accuracy (Reading 72%, Derby 57%, and Javiana 9%). Overall, these accuracy results are similar to those observed across the comparably well-represented monophyletic serovars (*P* = 0.11, two-sided Wilcoxon rank sum test).

### Algorithm performance in *E. coli*

**Fig 6:**
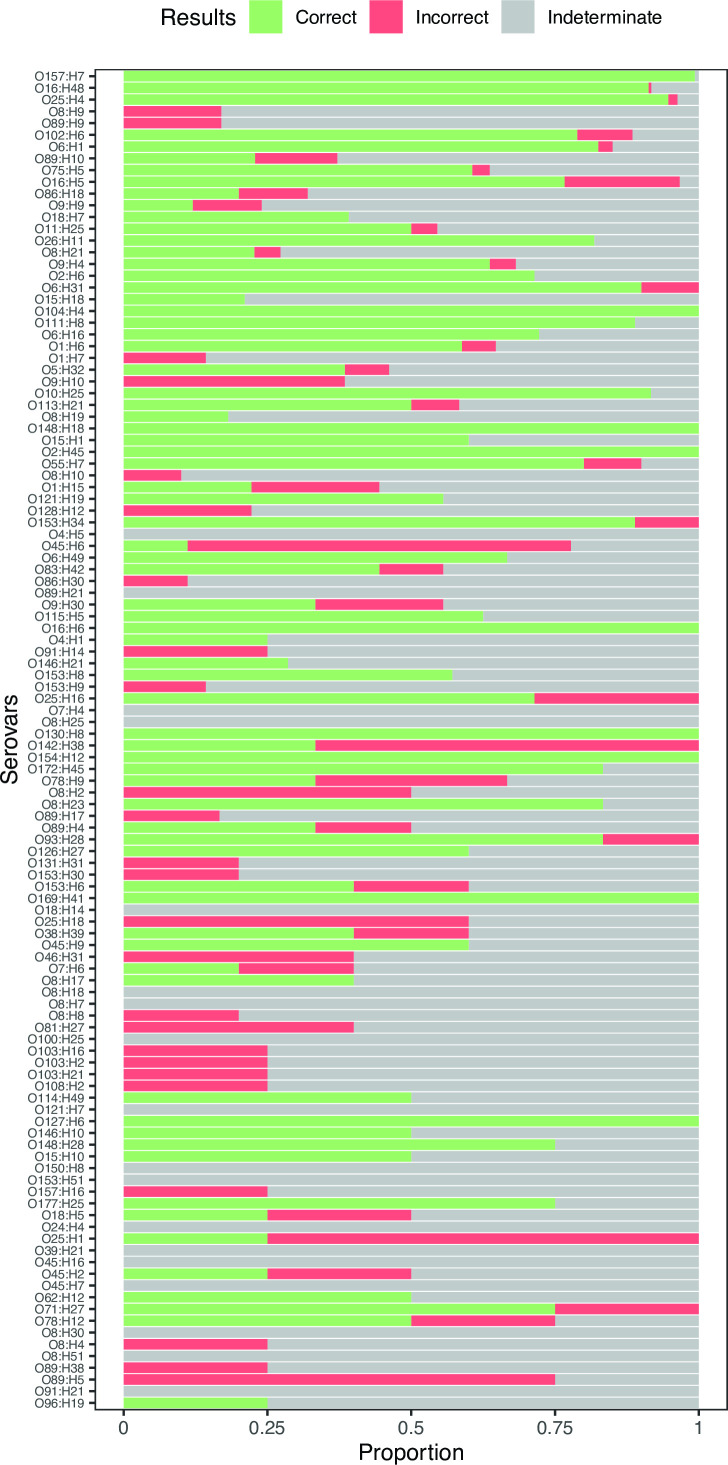
Serovar prediction accuracy in *E. coli* varies across serovars. The proportion of query sequences that resulted in correct, incorrect, or indeterminate (Materials and Methods) serovar assignments from a subset of 114 serovars with at least four entries in our *E. coli* reference database. Serovars are ordered from most representation (O157:H7, 284 assemblies) to least representation (O96:H19; four assemblies). Assemblies with ambiguously assigned O antigens were excluded from this figure. Rates of indeterminate serovar assignment were much higher in *E. coli* than in *Salmonella*.

We similarly evaluated our serovar prediction method in *E. coli* (Fig. 6) and found that it was effective for some serovars but had a much higher rate of indeterminate assignments and a larger dropoff in accuracy for less-represented serovars than we observed in *Salmonella*. In the *E. coli* serovars with the most representation in our reference database (100+ assemblies), we made correct serovar calls in 95.6% of test queries, with a 3.8% indeterminate rate. After that, the rate of correct predictions dropped substantially. In moderately represented serovars (20–99 assemblies in the reference), our correct prediction rate was 48.9% with a 42.2% indeterminate rate. In serovars with 10–19 assemblies in the reference, our correct prediction rate was 46.2% with a 45.4% indeterminate rate. Serovar prediction accuracy was worst in the serovars with the least representation in the reference database (4–9 assemblies). In this group, the method had a correct prediction accuracy of 30.0% with a 56.4% indeterminate rate. Incorrect predictions remained low to modest (<14%) across all reference-size groups (Table 2).

**TABLE 2:** Serovar prediction accuracy in *E. coli* serovars depends on the amount of representation in our reference database^*a*^

	Correct	Incorrect	Indeterminate	Number in group
100–300 assemblies	95.6%	0.6%	3.8%	3
20–99 assemblies	48.9%	8.9%	42.2%	18
10–19 assemblies	46.2%	8.4%	45.4%	20
4–9 assemblies	30.0%	13.6%	56.4%	83

^*a*^ Percentage correct, incorrect, and indeterminate rates are averages across serovars stratified into groups by the number of genomes a serovar has in the reference database.

In order to understand the difference in observed accuracy between *Salmonella* and *E. coli* serovar predictions, we further investigated the diversity and phylogenetic structure of serovars in each species. The *Salmonella* phylogeny contained 52 serovars containing at least four assemblies, while the *E. coli* phylogenetic tree contained 124 serovars containing at least four assemblies. It is possible that the increased variety of serovars in *E. coli* in combination with the much larger total phylogenetic tree makes it more difficult to make accurate serovar predictions. However, we suspect that *E. coli* serovars that do not form phylogenetically coherent clade(s) may also contribute to lower overall performance. In large *E. coli* serovars (20+ reference assemblies in our phylogenetic tree), we observed a polyphyletic clade structure in six serovars. Among these six large polyphyletic *E. coli* serovars, we had a poor prediction rate (<30% correct) in four: O8:H9, O89:H9, O9:H9, and O8:H21, which was significantly lower than the prediction accuracy across comparably well-represented monophyletic serovars (*P* = 0.03, two-sided Wilcoxon rank sum test).

The serovar prediction accuracy of our method varied substantially among *E. coli* serovars, with notable differences in accuracy among O157 and the other “big six” *E. coli* serovars especially relevant to foodborne disease. Our method had a very high correct prediction rate (99.3%, *n* = 284) in the highly relevant *E. coli* pathogenic serovar O157:H7. Our method also had a high correct prediction rate in O26 (81.8%, *n* = 22) and O111 (88.9%, *n* = 18). The correct prediction rate was lower for O45:H9 (60%, *n* = 5) and O121:H19 (55.6%, *n* = 9), and our method did not effectively predict (0%, *n* = 12) in O103. There were no O145 reference entries in our data set.

### Serovar prediction on environmental samples

We evaluated the performance of our method on a set of *Salmonella* bacterial isolates collected from various environmental sources including human stool, animal waste, and lairage swabs (Table 3). We performed amplicon sequencing of the full-length 16S rRNA gene and WGS on 10 isolates obtained from environmental samples. In each sample, we identified a full profile of seven 16S rRNA gene alleles that were then used by our method to predict the serovar. We also made serovar predictions from the whole-genomes sequences as ground truth (Materials and Methods). For 5 of the 10 environmental isolates, identical serovars were predicted based on our method and WGS. In three other isolates from serovars that were present in our reference database, our 16S-based method made two indeterminate predictions and one incorrect prediction. Two isolates were not able to be predicted with our method because the WGS predicted that serovar (Sundsvall) was not present in our reference data. Notably, we made accurate predictions for the highly relevant foodborne pathogen serovars enteritidis, typhimurium, and monophasic typhimurium.

**TABLE 3:** Results of serovar prediction on *Salmonella* bacterial isolates^*a*^

Our prediction (% in clade)	WGS prediction	Result	Source	Accession no.
Enteritidis (99%)	Enteritidis	Correct	Human stool	SAMN11478315
Enteritidis (99%)	Enteritidis	Correct	Not listed	SAMN15508318
Typhimurium/monophasic (100%)	Typhimurium	Correct	Vegetative buffer	PDT000640020.1
Typhimurium/monophasic (99%)	Typhimurium	Correct	Human stool	PDT000465258.1
Enteritidis (99%)	Agona	Incorrect	Human stool	PDT000550769.1
Indeterminate	Muenster	Indeterminate	Sheep feces	PDT000688730.1
Typhimurium/monophasic (99%)	Monophasic Typhimurium	Correct	Lairage swab	SAMN13663197
Indeterminate	Sundsvall	Not in tree	Caprine feces	SAMN13513401
Indeterminate	Typhimurium	Indeterminate	Caprine cecal content	PDT000641676.1
Quebec/Soahanina (50%/50%)	Sundsvall	Not in tree	Caprine feces	PDT000641787.1

^*a*^ We performed full-length 16S rRNA gene amplicon sequencing on a set of isolates for which WGS was already available. We report the prediction from our algorithm, the WGS serovar prediction, and the result of our method’s prediction treating the WGS prediction as the ground truth.

We also performed amplicon sequencing of the full-length 16S rRNA gene directly on 24 samples collected from retail meat rinsates, i.e., without first obtaining isolates. Of these 24 samples, 8 samples contained significant abundances of amplicon sequencing variants (ASVs) assigned to *E. coli* species, and 2 samples contained traces of *E. coli* but did not have sufficient sequencing depth to identify more than 1 ASV. *Salmonella* was not detected in any of the samples. We were able to clearly determine the full seven-allele rRNA gene profile in three of the eight samples with significant *E. coli* abundance. We ran serovar predictions using our algorithm on these three samples and predicted that one sample contained *E. coli* serovar 0157:H7 and the other two resulted in an indeterminate result. The remaining five samples with significant *E. coli* abundances appeared to contain multiple strains in comparable abundances, and we were unable to unambiguously deconvolve the different strains’ 16S rRNA gene profiles.

## DISCUSSION

Here, we presented a computational tool that can make serovar-level assignments in *Salmonella* and *E. coli* from full-length 16S rRNA gene sequence data. Conventional wisdom might state that 16S rRNA gene sequencing can only achieve species-level resolution, but we think our results here make clear that subspecies resolution is possible from the kind of full-length high-accuracy sequencing now available. Unique aspects of our approach that enabled taxonomic resolution well below species level were our utilization of the full set of alleles from the multicopy 16S rRNA gene and the use of phylogenetic placement into a serovar-labeled reference database. Our method was accurate for the prediction of *Salmonella* serovars, was less accurate in *E. coli* generally but showed potential for prediction of pathogenic *E. coli* serovars from the “big six,” and is in principle generalizable to any other bacterial species. Due to the phylogenetic nature of our prediction algorithm, it depends on high-quality and extensive genomic reference sequence data to construct appropriate references for the bacterial species of interest. *Salmonella* and *E. coli* were a good starting point to develop our method because of the large quantity of reference data, with over 500,000 reference entries in *Salmonella* and 300,000 entries in *E. coli* available for download from the GenomeTrakr database. Our approach can easily be translated to other bacterial species with a phylogenetically coherent subspecies classification schema by constructing a new phylogenetic tree for the organism of interest and assembling a reference 16S rRNA gene sequence database. Potential candidate bacteria for future application include *Staphylococcus aureus*, *Campylobacter jejuni*, *Klebsiella pneumoniae*, and *Listeria monocytogenes*.

The number of high-quality reference genomes available for a serovar was strongly correlated with our ability to make accurate predictions. Our method achieved its highest accuracies in serovars with a large representation in our database for both *Salmonella* and *E. coli* (100+ assemblies). It is important to note that the number of genomes that could serve as effective references for our method was much smaller than the raw number present in many WGS databases because we required genomes that fully resolved the multiple copies of the ribosomal operon. Resolving repeat elements like the rrn operon is a classic challenge for short-read sequencing, and most of the assemblies that qualified for our reference databases included long-read sequencing data. The GenomeTrakr database is being rapidly expanded, with over 5,000 new entries being submitted every month, but the rate of increase in high-quality genomes with resolved repeat elements is much smaller.

To our knowledge, our method is the first one that leverages the full complement of alleles from the 16S rRNA marker gene to improve the resolution of taxonomic classification, although some previous studies have provided case-study demonstrations (19–21). Amplicon sequencing randomly samples from the multiple copies of the 16S rRNA gene present in bacterial genomes, and variation between those alleles is increasingly evident when sequencing the full-length gene. Instead of considering this allelic variation just a nuisance, we chose to take advantage of this information by leveraging the data from the full set of 16S rRNA gene sequences for classification. It was common for two serovars to share a portion of identical 16S rRNA gene alleles but have unique differences in other alleles. Our method considers the unique nucleotide differences on a per-allele basis, meaning we can distinguish very similar isolates with nucleotide changes in specific 16S rRNA gene copies. The accuracy and length of amplicon sequencing with modern long-read sequencing platforms like Pacbio HiFi and Element Biosciences LoopSeq make this approach feasible (18, 19, 22).

Evaluating whether a taxonomic classification method is of sufficient accuracy for specific applications is an important but difficult question that probably needs to meaningfully consider accuracy metrics on a per-taxon basis (23). Our method was only modestly accurate for predicting *E. coli* serovars in general: Roughly 40% of *in silico* test queries returned an indeterminate serovar classification, and 2/3 of the community samples for which we extracted an *E. coli* 16S rRNA gene profile also resulted in indeterminate classifications. However, the classification accuracy for the notorious O157:H7 serovar was exceptionally high for *in silico* testing, and O157:H7 was positively identified directly from amplicon sequencing in one of the community samples. If a specific application can accept a high rate of indeterminate serovar assignments for other *E. coli* serovars but needs sensitive and specific detection of O157:H7, our method may be appropriate. More generally, if there is a defined set of species or subspecies taxa whose identification is critical to the mission, then the accuracy of classification methods should focus primarily on those taxa over broad cross-taxa averages (23).

A weakness of our method and its envisaged application to amplicon sequencing from complex samples is its dependence on having the complete 16S rRNA gene profile as a query. This weakness is most acute in complex environmental samples, where strain confusion and low relative abundance can hamper the recovery of the full 16S rRNA gene profile from specific strains present in the sample. For example, in only 3/8 of the environmental samples considered here with significant *E. coli* relative abundance, could we unambiguously determine a single strain profile from the mixture of *E. coli* 16S rRNA gene sequences? Advancing our approach to work with partial queries containing only some of a strain’s alleles is one path for improvement. Technological improvements in the cost, length, and throughput of high-accuracy sequencing will also help. For example, sequencing larger portions of the rrn operon including some or all of the ITS and 23S genes may allow for serovar-level classification from single amplicon sequences (22, 24), largely side-stepping the strain confusion problem. But key limitations will likely remain including a limit of detection based on relative abundance within the community of organisms being amplified.

The cost of the methodology we are proposing for the identification of serovars in isolates (barcoding) and in mixed community samples (metabarcoding) is critical to its practical importance. The price of traditional serotyping from isolates via molecular methods has been estimated to cost on average $42.37 per sample (9, 25). WGS-based serotyping has been estimated to cost an average of $83.15 per sample, generally running at a higher cost than molecular serotyping (25). In the context of barcoding, i.e., molecular characterization from isolates, long-read amplicon sequencing costs of the full-length 16S rRNA gene are regularly advertised by Pacbio at $10 per sample, and more aggressive multiplexing with per-sample read targets of 200–300 reads per sample (sufficient to measure an isolate’s full 16S rRNA gene profile) could approach costs as low as $2 per sample. However, those very low per-sample costs depend critically on high levels of multiplexing, and thus, the need to regularly barcode hundreds or thousands of isolates. LoopSeq sequencing costs as performed for this manuscript were $90 per sample for bacterial isolates; however, this was based on a target read-depth of 1,000 reads per sample and sequencing-as-a-service at a small scale (<50 samples). We expect that LoopSeq barcoding costs would also scale down with lower read-depth targets that allow much greater multiplexing. In addition, recent events such as the acquisition of Loop Genomics by Element Biosciences and the concomitant shift in underlying sequencing technology will also affect the costs of LoopSeq for long-read amplicon sequencing going forward. The financial aspects of applying our approach to metabarcoding samples are more difficult to ascertain, but it is fair to say that as the number of samples in which large amounts of nontarget DNA might be present (such as food samples), the relative advantage of a targeted method like amplicon sequencing over untargeted sequencing increases.

Although real challenges exist to realizing the full potential of long-read amplicon sequencing of the 16S rRNA gene (and beyond), there is a clear value proposition going forward. Amplicon sequencing is an extremely effective approach for focusing sequencing on a taxonomic range of interest in the presence of large amounts of nontarget DNA. New sequencing technologies are changing what is possible from standard methods like amplicon sequencing. This includes 16S rRNA gene sequencing, a specific (to prokaryotes) but broad (to bacteria) approach to characterizing complex microbial communities that are being supercharged by the high-accuracy long-read sequencing technologies that have been developed and are becoming increasingly available. As reference databases and sequencing technologies continue to progress, there are exciting new possibilities, including effective subspecies-level classification from amplicon sequencing of the 16S rRNA gene across large swathes of pathogenic and nonpathogenic bacterial species.

## Data Availability

Reproducible analysis of computational code and generation of figures and data can be viewed at https://github.com/Dogrinev/Seroplacer_Manuscript. Analyses are available in the form of an HTML containing all outputs or R markdown files to re-run all code. Raw sequence data for environmental samples and bacterial isolates analyzed by amplicon sequencing can be found in the Sequence Read Archive (SRA) available at Bioproject ID PRJNA988246.

## SUPPLEMENTAL MATERIAL

Supplemental material msystems.00757-23-s0001.pdf Supplemental figure and table.

ASM does not own the copyrights to Supplemental Material that may be linked to, or accessed through, an article. The authors have granted ASM a non-exclusive, world-wide license to publish the Supplemental Material files. Please contact the corresponding author directly for reuse.
